# Rapid Identification and Differential Detection of Infectious Bursal Disease Virus by an Innovative ARMS‐qRT‐PCR Assay

**DOI:** 10.1155/tbed/2178256

**Published:** 2026-09-23

**Authors:** Weiwei Wang, Yuanzheng Qiao, Jiafeng Wu, Ruiyang Kong, Yan Zhang, Jun Shi, Jinnan Chen, Zhiyuan Wang, Huimin Zhao, Tianchao Wei, Teng Huang, Jianni Huang, Meilan Mo, Xiumiao He, Ping Wei

**Affiliations:** ^1^ Institute for Poultry Science and Health, Guangxi University, Nanning 530004, China, gxu.edu.cn; ^2^ Institute of Animal Husbandry and Veterinary Medicine, Fujian Industry Technology Innovation Research Academy of Livestock and Poultry Diseases Prevention and Control, Fujian Academy of Agricultural Sciences (FAAS), Fujian Key Laboratory for Control and Prevention of Avian Diseases, Fuzhou 350013, China, faas.cn; ^3^ Guangxi Key Laboratory for Polysaccharide Materials and Modifications, School of Marine Sciences and Biotechnology, Guangxi University for Nationalities, Nanning 530008, China, gxun.edu.cn

**Keywords:** ARMS-qRT-PCR, coinfection, differential detection, epidemiological surveillance, infectious bursal disease virus, rapid identification

## Abstract

Infectious bursal disease (IBD), caused by IBD virus (IBDV), is a major threat to the poultry industry, with the cocirculation of multiple genotyping strains complicating the diagnosis and control. Since the differences of antigenicity among different genotyping and their differences with the commonly used vaccine strains, rapid and accurate identification and differential detection are crucial for surveillance and control of IBDV. In this study, a novel amplification refractory mutation system quantitative reverse transcription PCR (ARMS‐qRT‐PCR) assay, for the rapid differential diagnosis of very virulent IBDV (vvIBDV), novel variant IBDV (nvIBDV), and attenuated IBDV (attIBDV) strains that are now popularly cocirculating in China and some other countries, was developed. By combining ARMS technology with qRT‐PCR, this method enables accurate differentiation of these genotypes, including the coinfected samples. The developed ARMS‐qRT‐PCR assay demonstrated high repeatability and specificity, with no cross‐reactivity among the three genotyping or other common avian pathogens. The assay exhibited high sensitivity and excellent linear correlation, with detection limits of 65.6, 57.8, and 70.2 copies/μL for vvIBDV, nvIBDV, and attIBDV, respectively. It also showed results that were completely consistent with the sequencing validation in identifying single and coinfected field samples. Crucially, it successfully identified and quantified individual differential detection in complex dual and triple coinfection cases. This high‐throughput, user‐friendly method offers significant advantages over traditional techniques, making it an ideal tool for real‐time diagnosis, epidemiological surveillance, and outbreak management of IBDV.

## 1. Introduction

Infectious bursal disease (IBD) is a globally prevalent and highly contagious immunosuppressive disease that primarily affects young chickens [1] and, caused by IBD virus (IBDV), predominantly destroys B lymphocytes in the bursa of Fabricius [2]. Characterized by immunosuppression after the infection and the subsequenced infections with other pathogens [3, 4], IBD causes significant economic losses to the worldwide poultry industry. Although the clinical manifestations are short‐lived, the damage to the bursa is irreversible. The IBDV genome consists of two segments, A and B [5], which collectively encode five viral proteins: the RNA‐dependent RNA polymerase VP1, the capsid protein VP2, the scaffolding protein VP3, the protease VP4, and the nonstructural protein VP5. Two serotypes have been identified: serotype I (pathogenic to chickens) and serotype II (nonpathogenic to chickens) [6]. It has been confirmed that vvIBDV, attIBDV, and nvIBDV are currently circulating in China, with vvIBDV and nvIBDV being the dominant epidemic strains [4, 7, 8]. Like other segmented RNA viruses, different pathogenic types of IBDV can cocirculate in the field [9, 10]. Long‐term coinfections may provide potential conditions for the rapid evolution of the virus, posing a major challenge for control of this disease. Since the differences of antigenicity among different genotypes and their differences with the commonly used vaccine strains, it is crucial to develop a rapid method for the identification and genotyping of the currently circulating vvIBDV, nvIBDV, and attIBDV strains.

In addition to pathotype‐based classification, a new genotypic classification and nomenclature system based on the sequences of segments A and B sampled worldwide has recently been proposed. This system identified 10 well‐supported genogroups for segment A, including the novel genogroup reported in Portugal by Legnardi et al. [11], and five genogroups for segment B [7, 12, 13]. This genotypic framework provides a more comprehensive understanding of the genetic diversity and evolutionary relationships of IBDV and further highlights the genetic complexity of the currently circulating IBDV populations. Therefore, reliable and rapid molecular methods capable of distinguishing epidemiologically relevant IBDV genotypes remain essential for field surveillance and disease control.

With the cocirculation of multiple genotypes of IBDV, differential detection has become a critical process for characterizing and monitoring important viral strains. Currently, several molecular detection methods, such as RT‐PCR‐Seq, multiplex RT‐PCR, TaqMan qRT‐PCR, RT‐PCR‐RFLP, HRM‐qRT‐PCR, gene chips, nucleic acid hybridization, and digital PCR, have been developed for identification and genotyping of IBDV. While conventional sequencing analysis can identify the exact mutation sites of viruses, the process is time‐consuming and costly. For detecting and differentiating between field and vaccine strains of IBDV, a strain‐specific multiplex RT‐PCR technique was developed [14]. Furthermore, a real‐time TaqMan qRT‐PCR method was designed specifically to distinguish the domestically prevalent nvIBDV from non‐nvIBDV strains, yet it overlooked the dominant vvIBDV strains [15]. TaqMan qPCR offers high specificity and multiplexing capacity, and it requires costly fluorescent probes and complex optimization, which may limit its routine application in large‐scale surveillance and resource‐limited laboratories. RT‐PCR‐RFLP differentiates IBDV field strains from attenuated vaccine strains by digesting the viral genome with different restriction enzymes (e.g., *BstN*I, *Mbo*I, *Sac*I, *Ssp*I, *BamH*I, *Pst*I, *Sty*I, *Hha*I, *Stu*I, and *BspM*I) [16–18]. However, this method is not only complex and time‐consuming but also relies on enzyme cleavage sites that can be unreliable, sometimes difficulties in interpreting some results. As for other technologies such as gene chips, nucleic acid hybridization, and digital PCR, high cost and operational complexity remain significant barriers to their widespread application. The recently developed HRM‐qRT‐PCR assay by our group enables accurate identification and pathotyping of vvIBDV, nvIBDV, and attIBDV. However, because it relies on a single primer pair and melting‐curve analysis, it is unable to independently quantify the individual viruses in coinfected samples [19].

**Figure 1:**
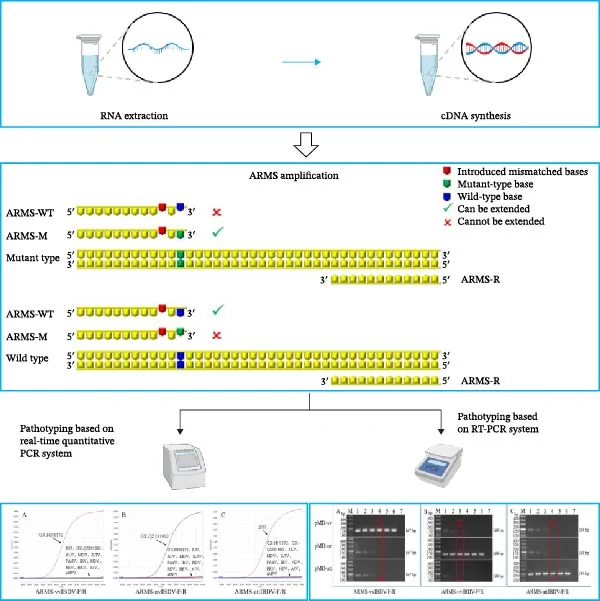
Scheme of the process of the ARMS‐RT‐qPCR assay for rapid identification and genotyping of IBDV.

While existing methods have greatly facilitated IBDV genotyping, the recent development of the amplification refractory mutation system PCR (ARMS‐PCR) offers a groundbreaking approach for designing highly differential, rapid, and reliable genotyping assays [20, 21]. This technique determines the genotype directly by electrophoresing the amplification products and removes the need for sequencing. Its numerous advantages, such as low cost, high throughput, operational simplicity, and closed‐tube operation that eliminates cross‐contamination, have led to its widespread use in diagnostics. In this study, we innovatively combined ARMS technology with quantitative RT‐PCR to develop an amplification refractory mutation system quantitative reverse transcription PCR (ARMS‐qRT‐PCR) for IBDV detection (Figure 1). This integration promotes the rapid and accurate diagnosis and genotyping of IBDV, significantly enhances clinical diagnostic efficiency, and provides a valuable tool for disease surveillance and control.

## 2. Materials and Methods

### 2.1. Viruses, Propagation, and Titer Determination

Infectious bronchitis virus (IBV), Newcastle disease virus (NDV), reticuloendotheliosis virus (REV), avian hepatitis E virus (aHEV), avian influenza virus (AIV), avian metapneumovirusa (aMPV), avian leukosis virus (ALV), Marek’s disease virus (MDV), infectious laryngotracheitis virus (ILTV), fowl adenovirus (FAdV‐4), and three genotypes of IBDVs (vvIBDV GX‐NN1172, nvIBDV GX‐QZ191002, and attenuated vaccine virus B87) were all preserved by our laboratory and were used in this study for specific validation.

### 2.2. DNA/RNA Extraction and cDNA Synthesis

Total DNA/RNA was extracted from the viruses using the spin column animal total RNA/DNA purification kit (Sangon Biotech, China) according to the manufacturer’s instructions. RNA concentration was assessed using the BioDrop ultramicro spectrophotometer (BioDrop, UK). For RNA viruses, 2 µg of RNA was reverse transcribed into cDNA using the All‐In‐One 5 × RT Master Mix Kit (ABM, Canada) following the established protocol in previous studies [22]. To ensure consistent evaluation conditions, all tested pathogens underwent the same nucleic acid extraction procedure. RNA viruses were subjected to the same reverse‐transcription protocol, whereas DNA viruses were directly used as templates for subsequent amplification.

### 2.3. Primer Screening, Design, and Evaluation

A total of 442 complete IBDV genome sequences containing sufficient segment A sequence information for alignment and mutation screening were retrieved from the NCBI database (GenBank numbers of representative strains: GQ451330, MW245064, AY444873, NC004178, AF092943, D49706, JX134483, MZ066613, MN485882, MN393076, MT179720, MT179721, X84034, AF194428, AF499929, DQ906921, DQ403248, and AJ310185). The polymorphic sites targeted in this study were selected because they harbor stable, genotype‐associated nucleotide signatures that reliably differentiate vvIBDV, nvIBDV, and attenuated strains. Extensive alignment and mutation screening analyses confirmed that these positions exhibit high intragroup conservation while containing distinct intergroup variations, thereby representing optimal targets for ARMS primer design. Accordingly, three pairs of ARMS‐specific primers targeting vvIBDV, nvIBDV, and attIBDV, respectively, were designed (Table 1). Each primer pair contains a discriminatory 3′‐terminal nucleotides that is perfectly complementary to the respective nucleic acid bases of vvIBDV, nvIBDV, and attIBDV, enabling efficient amplification of target fragments from each genotype without amplifying other variants. The specificity of the designed primers was preliminarily evaluated using the NCBI Primer‐BLAST tool, and no significant secondary structures and dimers were evaluated using Oligo 7. The primers were further synthesized by BGI Technology Co., Ltd., Beijing, China.

**Table 1:** The primer sequence of the ARMS‐qRT‐PCR assays.

Target pathotypes	Primers	Primer sequence (5′→3′)	Primer binding site (bp)	Product length (bp)
Universal	IBDV‐F	TGTCCTCAGCTTGCCCACA	625–643	855
	IBDV‐R	GCCCGAATTATGTCTTTGAAGCC	1457–1479	
vvIBDV	ARMS‐vvIBDV‐F	ATGATCTGGGGTATGTGAGACGC	648–670	167
	ARMS‐vvIBDV‐R	TGTAATTGTCACTCCACCTGC	794–814	
nvIBDV	ARMS‐nvIBDV‐F	GGCTTGTTGACCCCATACTT	666–685	149
	ARMS‐nvIBDV‐R	TGTGATTGTTACCCCACCTGT	794–814	
attIBDV	ARMS‐attIBDV‐F	AATTCTCATCACAGTACCACC	774–794	184
	ARMS‐attIBDV‐R	ACAGCCCTGGTGATTACAGTT	937–957	

### 2.4. Recombinant Standard Plasmid Construction and Sequencing

Recombinant standard plasmids (pMD‐vv, pMD‐nv, and pMD‐att) were constructed by amplifying the cDNA of strains GX‐NN1172, GX‐QZ191002, and B87 with universal primers IBDV‐F/IBDV‐R (Table 1), followed by cloning the amplicons into the pMD18‐T vector and verifying them via Sanger sequencing (BGI, Guangzhou, China). Plasmid concentrations were determined using a BioDrop ultramicro spectrophotometer (BioDrop, England), and copy numbers were calculated using the formula: copies/μL = [6.022 × 10^23^ × concentration (g/μL)]/(plasmid base pairs × 660) [23].

### 2.5. ARMS‐qRT‐PCR Protocol and Standard Curve

To optimize the reaction conditions of the ARMS‐qRT‐PCR assay, different annealing temperatures (54, 56, 58, 60, 62, and 64°C) were tested in a total reaction volume of 20 μL to evaluate the specificity of ARMS primers among vvIBDV, nvIBDV, and attIBDV. The reaction mixture consisted of 10 μL of 2× ChamQ Universal SYBR qPCR Master Mix, 0.4 μL of forward primer (10 μM), 0.4 μL of reverse primer (10 μM), 2 μL of template DNA, and 7.2 μL of ddH_2_O. The thermal cycling protocol was as follows: predenaturation at 95°C for 3 min; 40 cycles of 95°C for 10 s, 58/60°C for 30 s, and 72°C for 30 s; followed by a melting curve analysis at 95°C for 10 s, 65°C for 60 s, and 97°C for 1 s. Each ARMS‐qRT‐PCR reaction contains a single group–specific primer pair and is designed to specifically detect one target IBDV group (vvIBDV, nvIBDV, or attIBDV). Based on the optimized system and conditions, standard curves were generated using 10‐fold serial dilutions (10^8^–10^1^ copies/μL) of the recombinant standard plasmids.

### 2.6. Analytical Specificity Assay

To evaluate the specificity of ARMS‐qRT‐PCR in detecting different genotypes of IBDV, common avian infectious disease pathogens, including IBV, NDV, REV, aHEV, AIV, ALV, MDV, ILTV, and FAdV‐4, were tested simultaneously according to the abovementioned optimized ARMS‐qRT‐PCR protocol. ddH_2_O was used as the negative control.

### 2.7. Analytical Sensitivity Assay and Standard Curves

To evaluate the sensitivity of ARMS‐qRT‐PCR reactions, positive standard plasmids were 10‐fold serial dilutions with concentrations ranging from 6.56 × 10^10^ to 6.56 × 10^0^ copies/μL for pMD‐vv (5.78 × 10^10^ to 5.78 × 10^0^ copies/μL for pMD‐nv and 7.02 × 10^10^ to 7.02 × 10^0^ copies/μL for pMD‐att) using ARMS primers, respectively, to determine the detection limits of ARMS‐qRT‐PCR assays in the identification and differential detection of IBDV. Each dilution level was tested in triplicate across three independent experimental runs.

### 2.8. Analytical Repeatability Assay

Three standard plasmids were serially diluted 10‐fold and used as templates to test intra‐ and interassay repeatability and stability of the newly developed ARMS‐qRT‐PCR method. Three independent serial dilution experiments were performed to determine the limit of detection (LoD). Each dilution level was tested in triplicate, and the LoD was defined as the lowest concentration at which all three replicate reactions consistently produced positive amplification results.

### 2.9. Differential Detection of IBDV in Experimentally Infected Chickens and Clinical Samples by ARMS‐qRT‐PCR

A coinfection study was conducted on 40 nonvaccinated three‐yellow chickens (28‐day‐old) randomly divided into eight groups (*n* = 5), and the health status and vaccination history of the birds were checked for control before infection. Groups were assigned to different infection protocols: single infections with NN1172 (Group A), B87 (Group B), and QZ191002 (Group C); dual infections with NN1172 + B87 (Group D), NN1172 + GX‐QZ191002 (Group E), and GX‐QZ191002 + B87 (Group F); and a triple coinfection with NN1172 + B87 + GX‐QZ191002 (Group G). Group H served as an uninfected control, receiving phosphate‐buffered saline (PBS). The birds were challenged orally with a dose of 10^5^ TCID_50_/0.5 mL of the respective IBDVs. At 7 days postinfection (35 days of age), all birds were euthanized, and bursa of Fabricius tissues were simultaneously detected and screened using the ARMS‐qRT‐PCR assay and the conventional nested RT‐PCR developed by Wei et al. [18]. In addition, the developed ARMS‐qRT‐PCR method was applied to detect five, two, and three samples of the vvIBDV, nvIBDV, and attIBDV infected cases, respectively, by our laboratory sequencing identification to determine its accuracy in discriminating different genotypes of IBDVs. The detection accuracy was evaluated based on the concordance of both the ARMS‐qRT‐PCR and the nested RT‐PCR + sequencing results.

## 3. Results

### 3.1. Unique Mutations in Different Genotype of the Representative IBDVs

**Figure 2:**
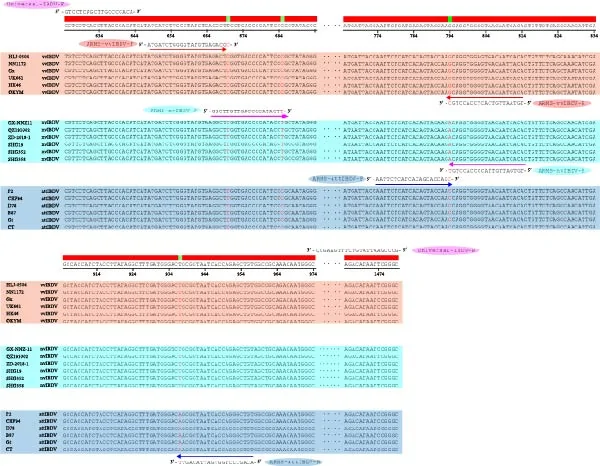
Unique nucleotide mutations of the ARMS‐RT‐qPCR amplification region between the different genotypes of IBDV.

By aligning and screening 442 full genome sequences, unique nucleotide mutations were identified at sites 670, 685, 794, and 937 in segment A of the genome of IBDVs. ARMS primers were then designed for these sites to target different genotype strains of IBDV (Figure 2).

### 3.2. Determination of the Annealing Temperature for ARMS Primers Targeting Different Genotypes

**Figure 3:**
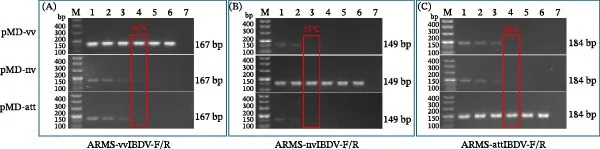
The optimal annealing temperatures of the ARMS primers were determined by RT‐PCR. Line 1–6: 54, 56, 58, 60, 62, and 64°C; line 7: negative control. (A) ARMS‐vvIBDV‐F/R; (B) ARMS‐nvIBDV‐F/R; and (C) ARMS‐attIBDV‐F/R.

The optimal annealing temperatures of the ARMS primers were determined by RT‐PCR (Figure 3). The results showed that the optimal annealing temperature for ARMS‐vvIBDV‐F/R was 60°C, at which only pMD‐vv was successfully amplified, while no amplification was observed for pMD‐att or pMD‐nv. For ARMS‐nvIBDV‐F/R, the optimal annealing temperature was 58°C, allowing specific amplification of pMD‐nv without any amplification of pMD‐att or pMD‐vv. Similarly, the optimal annealing temperature for ARMS‐attIBDV‐F/R was 60°C, under which only pMD‐att was amplified, while pMD‐vv and pMD‐nv showed no amplification.

### 3.3. Sensitivity and Standard Curve for ARMS‐qRT‐PCR

**Figure 4:**
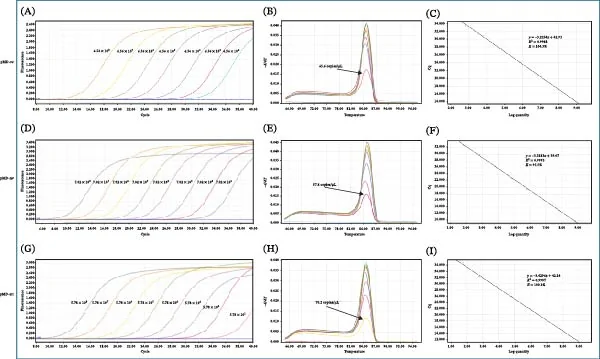
The sensitivity and standard curve for ARMS‐qRT‐PCR assay. (A, D, G) The sensitivity analysis of the 10‐fold dilutions of the three pathotypes plasmids. (B, E, H) The melting peak curves of the 10‐fold dilutions of the three pathotypes plasmids. (C, F, I) The standard curve of the 10‐fold dilutions of the standard plasmids of the three pathotypes.

To evaluate the sensitivity of the ARMS‐qRT‐PCR assay, pMD‐vv, pMD‐nv, and pMD‐att templates were tested over a dilution series from 10^10^ to 10^1^ copies/μL (Figure 4A,D,G). The positive detection rate was 100% at each dilution level tested. An excellent linear correlation was observed across this range (Figure 4C,F,I). The LODs are 65.6 copies/μL for pMD‐vv, 57.8 copies/μL for pMD‐nv, and 70.2 copies/μL for pMD‐att, confirming the high sensitivity of the assay (Figure 4B,E,H). The correlation coefficients (*R* ^2^) and amplification efficiencies (E) of the standard curve equations were as follows: vvIBDV strain (*y* = −3.2254*x* + 42.93, *R* ^2^ = 0.9988, and *E* = 104.3%); nvIBDV strain (*y* = −3.4296*x* + 42.14, *R* ^2^ = 0.9997, and *E* = 95.5%); and attIBDV strain (*y* = −3.3183*x* + 39.07, *R* ^2^ = 0.9993, and *E* = 100.1%). These results indicate a strong linear correlation between the initial template concentration and the Ct value (Figure 4C,F,I).

### 3.4. Specificity of the ARMS‐qRT‐PCR Analysis

**Figure 5:**
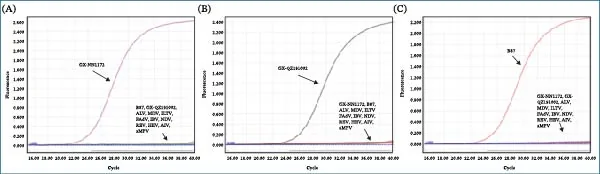
The specificity of the ARMS‐qRT‐PCR assay. ddH_2_O was used as the negative control. (A) ARMS‐vvIBDV‐F/R; (B) ARMS‐nvIBDV‐F/R; and (C) ARMS‐attIBDV‐F/R.

Under the optimized reaction conditions, the developed ARMS‐qRT‐PCR assay was applied to detect the DNA/cDNA from a panel of common avian pathogens. Amplification curves were observed only for IBDV strains NN1172, B87, and GX‐QZ191002 using the three specific ARMS primers. No amplification was detected for other common avian pathogens, including IBV, NDV, REV, aHEV, AIV, aMPV, ALV, MDV, ILTV, and FAdV‐4, or in the ddH_2_O control, demonstrating high specificity of the method for detecting the three different IBDV genotypes (Figure 5A–C).

### 3.5. Repeatability Test of ARMS‐qRT‐PCR Analysis

To evaluate the repeatability of the ARMS‐qRT‐PCR assay, intra‐ and interassay comparisons were performed using serial dilutions (ranging from 10^8^ to 10^4^ copies/μL) of the standard plasmids of three path types. The results showed that the coefficients of variation (CVs) for the Ct values of the three standard plasmids, pMD‐vv, pMD‐att, and pMD‐nv, were 0.21%–0.51% for the intra‐assay and 0.22%–1.15% for the interassay comparisons, indicating that the method has good repeatability (Table 2).

**Table 2:** The repeatability (intra‐ and interassay batch comparisons) of the ARMS‐qRT‐PCR assays, with each test repeated three times.

Plasmids	Concentration (copies/μL)	Intra‐assay		Interassay	
		Cq	CV/%	Cq	CV/%
pMD‐vv	6.56 × 10^6^	21.13 ± 0.11	0.51	20.94 ± 0.18	0.85
	6.56 × 10^4^	27.47 ± 0.06	0.20	27.45 ± 0.15	0.55
	6.56 × 10^2^	33.46 ± 0.11	0.33	33.55 ± 0.22	0.64
pMD‐nv	7.02 × 10^6^	16.28 ± 0.07	0.45	16.41 ± 0.13	0.82
	7.02 × 10^4^	22.62 ± 0.05	0.21	22.44 ± 0.05	0.22
	7.02 × 10^2^	29.91 ± 0.10	0.32	30.08 ± 0.13	0.43
pMD‐att	5.78 × 10^6^	19.08 ± 0.06	0.31	19.16 ± 0.22	1.15
	5.78 × 10^4^	25.48 ± 0.06	0.22	25.49 ± 0.15	0.57
	5.78 × 10^2^	32.26 ± 0.15	0.46	32.40 ± 0.33	1.03

### 3.6. Identification and Genotyping of IBDV in Experimentally Infected Chickens and Clinical Samples by ARMS‐qRT‐PCR

**Figure 6:**
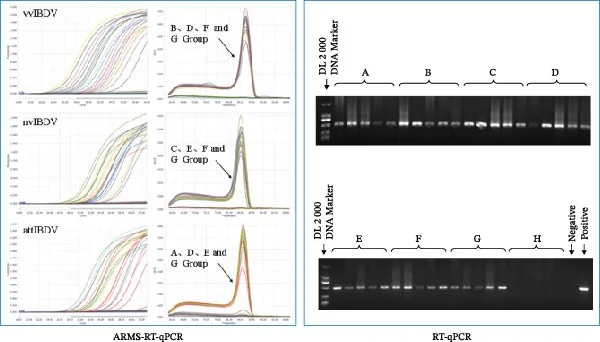
Evaluation of the artificial coinfection samples by ARMS‐qRT‐PCR and the conventional RT‐PCR assay. The single infection groups A, B, and C were individually challenged with NN1172, B87, and GX‐QZ191002, respectively. Groups D, E, and F were the double coinfected groups, with birds coinfected with NN1172 and B87, NN1172 and GX‐QZ191002, and GX‐QZ191002 and B87, respectively. Group G was triple coinfected with NN1172, GX‐QZ191002, and B87 simultaneously. Group H as the control group.

**Figure 7:**
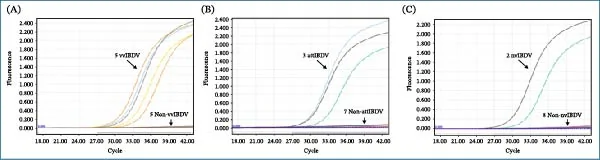
Evaluation the clinical samples by ARMS‐qRT‐PCR with the conventional RT‐PCR + sequencing assay. (A) ARMS‐vvIBDV‐F/R; (B) ARMS‐nvIBDV‐F/R; and (C) ARMS‐attIBDV‐F/R.

To further evaluate the performance of the ARMS‐qRT‐PCR assay for the differential detection of vvIBDV, nvIBDV, and attIBDV genotypes, 40 experimentally infected chicken samples representing single, dual, and triple IBDV infections were analyzed. The ARMS‐qRT‐PCR assay identified 35 positive and five negative samples, and all results were completely consistent with those obtained by conventional nested RT‐PCR, followed by sequencing (Figure 6). No false‐positive or false‐negative results were observed among the tested samples. In addition, 10 independent sequencing–confirmed samples, including five vvIBDV, two nvIBDV, and three attIBDV samples, were analyzed by ARMS‐qRT‐PCR. All 10 samples were correctly differentiated according to their corresponding IBDV genotypes, yielding complete agreement with the sequencing results (Figure 7). Collectively, these findings demonstrate that the ARMS‐qRT‐PCR assay exhibited high sensitivity, specificity, and overall concordance (100%) with the reference results for the differential detection of the three targeted IBDV genetic groups, including samples from single, dual, and triple infections.

## 4. Discussion

In 1991, vvIBDV was first reported in China and subsequently spread rapidly across the country, causing significant economic losses to the poultry industry [8]. In recent years, large‐scale outbreaks of nvIBDV infections have emerged in the poultry industry, mainly characterized by subclinical infections, making it difficult to accurately and early clinically diagnose these variant viruses [7, 24]. Moreover, the widespread use of live‐attenuaed vaccines in China has further complicated the field situation of IBDV infections and the diagnosis. Currently, vvIBDV, nvIBDV, and attIBDV have been confirmed as the three major genotypes of IBDV infections in China [4, 7, 8, 24–27]. The clinical symptoms and pathological changes caused by these genotypes are difficult to distinguish on‐site, particularly in cases of coinfection with different IBDV genotypes. So, it is essential to develop new molecular diagnostic and genotyping techniques that can accurately and rapidly distinguish and diagnose the three major epidemic strains of vvIBDV, nvIBDV, and attIBDV in clinical IBD outbreaks, which are crucial for effective disease surveillance, prevention, and timely control.

Accurate genotyping plays a critical role in detecting and controlling the spread of the emerging and high‐risk strains in the poultry industry, but traditional methods like RT‐PCR‐Seq, RT‐PCR‐RFLP [16], TaqMan qRT‐PCR [15], multiplex RT‐PCR [14], HRM‐qRT‐PCR [19], gene chips, nucleic acid hybridization, and digital PCR are expensive and/or time‐consuming, thereby limiting their application for real‐time surveillance of different IBDV genotypes. In contrast, ARMS‐qRT‐PCR provides a probe‐free, cost‐effective alternative with high discriminatory power, especially suitable for rapid screening and quantitative analysis of coinfected samples. In this study, we explored the combination of ARMS and qRT‐PCR technologies to develop an ARMS‐qRT‐PCR assay for identifying and distinguishing IBDV genotypes. Based on extensive screening of 422 sequences of segment A, three pairs of specific ARMS primers targeting vvIBDV, nvIBDV, and attIBDV, respectively, were designed (Figure 2), each demonstrating temperature‐dependent high specificity among the three genotypes (Figure 3). This design exhibits good reproducibility and simplicity, with the Ct values of intra‐assay under 0.51% and interassay under 1.15% (Table 2), making it well‐suited for the identification and genotyping of large‐scale field samples while maintaining high differentiation capability. In contrast to HRM‐qRT‐PCR [19], which relies on a single primer pair and melting‐curve discrimination and therefore cannot quantify individual viruses in coinfected samples, the ARMS‐qRT‐PCR employs allele‐specific 3‐terminal primers, enabling simultaneous discrimination and independent quantification of vvIBDV, nvIBDV, and attIBDV in coinfected samples.

The assay exhibited high sensitivity, with detection limits at 65.6, 57.8, and 70.2 copies/μL for vvIBDV, nvIBDV, and attIBDV, respectively (Figure 4B,E,H), outperforming the conventional RT‐PCR (~10^3^ copies/μL) though being slightly less sensitive than traditional dye–based qRT‐PCR (~10^1^ copies/μL) [18, 28]. Although ARMS‐RT‐qPCR is also based on a dye‐based qPCR system, its sensitivity is lower than that of the traditional dye–based qPCR, likely due to the introduction of mismatched bases at the 3′‐terminal of the primers, which reduces primer efficiency even for the target genotype. The entire amplification process of this method requires only 60 min, enabling high‐throughput analysis of 32 samples (including positive and no‐template controls) simultaneously in a single run. Unlike labor‐intensive traditional methods like RT‐PCR‐RFLP/Seq [16, 18], it presents distinct advantages for real‐time, large‐scale epidemiological surveillance.

This assay demonstrates high specificity, with no cross‐reactivity among different genotypes of IBDV or with other common avian pathogens (Figure 5). Furthermore, evaluation of 40 artificial coinfections and 10 clinical samples revealed a high concordance between the ARMS‐RT‐qPCR and the conventional RT‐PCR (Figures 6 and 7). The ARMS‐RT‐qPCR method successfully identified the three traditional genotypes of vvIBDV, nvIBDV, and attIBDV, as well as coinfection samples of vvIBDV + nvIBDV, nvIBDV + attIBDV, and vvIBDV + attIBDV. The results were fully consistent with those obtained by sequencing validation, even in complex triple coinfection samples. We explain that ARMS‐qRT‐PCR maintains specificity because each primer pair independently amplifies only its perfectly matched target, enabling accurate detection and quantification even in complex coinfection scenarios. Moreover, this method overcomes the limitations of traditional genotyping methods, enabling not only rapid viral genotyping but also facilitating quantitative analysis of viruses in samples, significantly improving the efficiency of clinical genotyping and outbreak surveillance and establishing it as a valuable tool for genotyping during IBD outbreak investigations.

## 5. Conclusion

In summary, this study is the first to develop an innovative ARMS‐RT‐qPCR assay for the rapid detection and genotyping of vvIBDV, nvIBDV, and attIBDV. It enables the accurate identification and discrimination of all single or coinfected samples, alongside the quantification of individual genotypes within coinfection cases. Furthermore, its user‐friendly workflow and rapid processing time make it particularly suitable for real‐time epidemiological surveillance during IBD outbreaks.

## Author Contributions

Writing – original draft preparation: Weiwei Wang. Literature and illustrations: Weiwei Wang, Yuanzheng Qiao, Jiafeng Wu, Ruiyang Kong, Yan Zhang, Jun Shi, Jinnan Chen, Zhiyuan Wang, and Huimin Zhao. Conceptualization and methodology: Weiwei Wang, Tianchao Wei, Teng Huang, Jianni Huang, and Meilan Mo. Writing – review and editing: Xiumiao He and Ping Wei.

## Funding

This study was funded by the Natural Science Foundation of Fujian Province (Grant 2025J08104), the National Natural Science Foundation of China (Grants 31660717, 32160824, and 31560706), the Guangxi Special Funding on Science and Technology Research (Grant AA17204057), and the Innovation Project of Guangxi Graduate Education (Grant YCBZ2022034).

## Disclosure

All authors contributed to the manuscript and approved the final manuscript.

## Ethics Statement

The animal experiment in this study was approved by the Animal Experimental Ethical Committee of Guangxi University (Approval Number GXU‐2021‐157).

## Conflicts of Interest

The authors declare no conflicts of interest.

## Data Availability

All data have been included in the main figures and tables within the article.
